# Ergot alkaloids contribute to virulence in an insect model of invasive aspergillosis

**DOI:** 10.1038/s41598-017-09107-2

**Published:** 2017-08-21

**Authors:** Daniel G. Panaccione, Stephanie L. Arnold

**Affiliations:** Division of Plant and Soil Sciences, Genetics and Developmental Biology Program, West Virginia University, Morgantown, WV 26506 USA

## Abstract

*Neosartorya fumigata* (*Aspergillus fumigatus*) is the most common cause of invasive aspergillosis, a frequently fatal lung disease primarily affecting immunocompromised individuals. This opportunistic fungal pathogen produces several classes of specialised metabolites including products of a branch of the ergot alkaloid pathway called fumigaclavines. The biosynthesis of the *N. fumigata* ergot alkaloids and their relation to those produced by alternate pathway branches in fungi from the plant-inhabiting Clavicipitaceae have been well-characterised, but the potential role of these alkaloids in animal pathogenesis has not been studied extensively. We investigated the contribution of ergot alkaloids to virulence of *N. fumigata* by measuring mortality in the model insect *Galleria mellonella*. Larvae were injected with conidia (asexual spores) of two different wild-type strains of *N. fumigata* and three different ergot alkaloid mutants derived by previous gene knockouts and differing in ergot alkaloid profiles. Elimination of all ergot alkaloids significantly reduced virulence of *N. fumigata* in *G. mellonella* (*P* < 0.0001). Mutants accumulating intermediates but not the pathway end product fumigaclavine C also were less virulent than the wild type (*P* < 0.0003). The data indicate that ergot alkaloids contribute to virulence of *N. fumigata* in this insect model and that fumigaclavine C is important for full virulence.

## Introduction

Ergot alkaloids are specialised metabolites derived from prenylated tryptophan and produced by several fungi representing different phylogenetic lineages and occupying different ecological niches^1–6^. Lysergic acid-based ergot alkaloids produced by fungi in the Clavicipitaceae, including the plant pathogen *Claviceps purpurea* and several *Epichloë* species that grow as mutualistic symbionts of grasses, have profoundly affected humanity as toxins in agricultural crops^5, 6^ and as the bases of pharmaceuticals used to treat dementia, migraines, Parkinson’s disease, and hyperprolactinemia^7–12^. The activities of these alkaloids stem from their abilities to act as agonists or antagonists at receptors for monoamine neurotransmitters including serotonin, dopamine, adrenaline, and noradrenaline. Ecologically the ergot alkaloids of the Clavicipitaceae have been shown to serve as feeding deterrents in mammals and insects and also have insecticidal activities^13, 14^.

**Figure 1:**
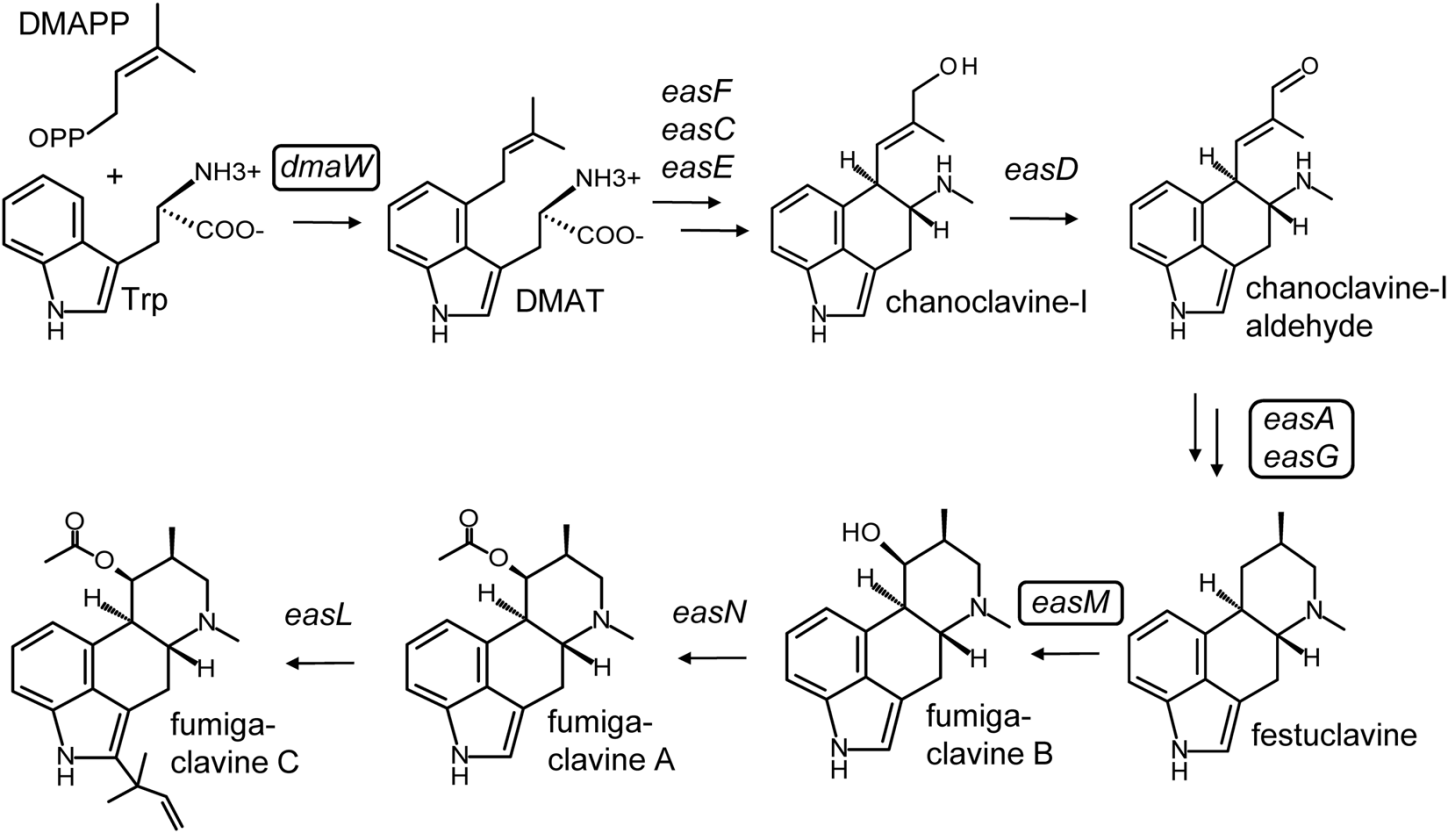
Important intermediates and end product of the ergot alkaloid pathway of *N. fumigata*. Genes controlling pathway steps are indicated, and genes knocked out in strains included in the present study are outlined. DMAPP, dimethylallylpyrophosphate; Trp, tryptophan, DMAT, dimethylallyltryptophan. Double arrows indicate additional, non-illustrated intermediates.

A different branch of the ergot alkaloid pathway leads to the production of fumigaclavines (Fig. 1) in certain members of the Trichocomaceae, including the opportunistic human and animal pathogen *Neosartorya fumigata* (*Aspergillus fumigatus*)^1–4^. Whereas several members of the Trichocomaceae produce representatives of the fumigaclavines, *N. fumigata* is the only fungus known to produce fumigaclavine C. The activities of the ergot alkaloids of the Trichocomaceae have not been studied as intensively as those of the Clavicipitaceae, but recent data demonstrate that fumigaclavine C inhibits tumor necrosis factor α production in human macrophages^15^ and leads to reduced expression of several other inflammatory cytokines in mice^16^. Moreover, fumigaclavine C is cytotoxic to cancer cells^17^.

*Neosartorya fumigata* is the most frequent cause of invasive aspergillosis, a disease that most frequently affects immunocompromised individuals and that has a high mortality rate^18–20^. Several lines of evidence indicate roles for specialised metabolites in virulence of *N. fumigata*, but direct evidence of the contribution of specific metabolites to virulence is scarce. In addition to its family of ergot alkaloids, *N. fumigata* produces gliotoxin, fumagillin, and fumitremorgins, among other specialised metabolites^19, 20^. Gene clusters for at least 13 metabolites, including the ergot alkaloids, are controlled by LaeA, a master transcriptional regulator of specialised metabolism^21^. LaeA-deficient mutants demonstrate reduced accumulation of several metabolites and have greatly reduced virulence in animal models^22, 23^. These data demonstrate that products of at least some LaeA-regulated genes are required for virulence and indicate roles for specialised metabolites in determining virulence. Among the metabolites produced by *N. fumigata*, gliotoxin has been most intensively studied because of its immunosuppressive activities^19, 20^. Tests of the contribution of gliotoxin to virulence in a murine model of invasive aspergillosis have yielded conflicting results, with the contribution of gliotoxin to virulence differing perhaps due to genetic background of the fungus or methods of suppressing host immunity^24–27^. *Neosartorya fumigata* also produces the polyketide-derived metabolite fumagillin, which suppresses the innate immune system of the model insect host *Galleria mellonella* rendering it significantly more susceptible to subsequent infection by the pathogen^28^.

A link between fumigaclavine C and virulence of *N. fumigata* can be hypothesised based on previous studies of peptide synthetases Pes1 and PesL. Whereas, *in vitro* studies indicate that reverse prenyl transferase FgaPT1 (also called EasL) is sufficient to convert fumigaclavine A to fumigaclavine C^29^, knockout studies indicate that peptide synthetases Pes1 and PesL also are required in some way for the conversion of fumigaclavine A to fumigaclavine C^30^. Pes1 and PesL mutants had additional altered phenotypes including increased accumulation of fumitremorgins and increased sensitivities to oxidative stress and antifungal azoles^30, 31^. Whether these additional phenotypes are associated with the loss of fumigaclavine C or with other activities of these peptide synthetases is unclear. Knockout of *pesL* reduced virulence in the insect model host *G. mellonella* ^30^. The role of Pes1 in virulence varied in two fungal strains in which mutants were made. Reeves *et al*.^31^ observed a significant reduction in virulence when *pes1* was knocked out in an Af293.1 background, but O’Hanlon *et al*.^30^ observed no difference in virulence when *pes1* was knocked out in an ATCC 46645 background.

Whereas mice have most frequently served as the model host in studies of infectious human pathogens, the larva of the greater wax moth *Galleria mellonella* has recently been used as a model host for pathogenicity assays of numerous mammalian pathogens^32–35^. In addition to being inexpensive and easy to maintain, experiments with this invertebrate do not require institutional oversight. In several studies, *G. mellonella* has been demonstrated to be a reliable and useful model of invasive aspergillosis^20, 28, 30, 31^. Results obtained with this invertebrate model correlate well with those obtained from tests of the same pathogens in mammalian models^33, 35, 36^. In one exception to this correlation, melanin-deficient mutants of *N. fumigata* had greatly reduced virulence in mice but increased virulence in *G. mellonella* ^37^.

Our previous research on the biosynthesis of ergot alkaloids in *N. fumigata* has resulted in the generation of gene knockout mutants that are deficient in the accumulation of some or all ergot alkaloids. Knockout of *dmaW*, the first gene in the ergot alkaloid pathway, eliminated all ergot alkaloids^38^ (Fig. 1). Blocking the pathway at the step controlled by *easA* prevented formation of festuclavine from chanoclavine-I aldehyde and resulted in accumulation of primarily the preceding intermediate chanoclavine-I^39, 40^ (also see Supplementary Information). Knockout of *easM* resulted in the accumulation of large quantities of festuclavine and prevented the formation of downstream fumigaclavines^41^ (Fig. 1). Our objective in this current study was to utilise these ergot alkaloid pathway mutants in virulence assays conducted with the larvae of *G. mellonella* to investigate the role of ergot alkaloids in virulence of *N. fumigata*.

## Results

### Contribution of ergot alkaloids to virulence of *N. fumigata* FGSC A1141

**Figure 2:**
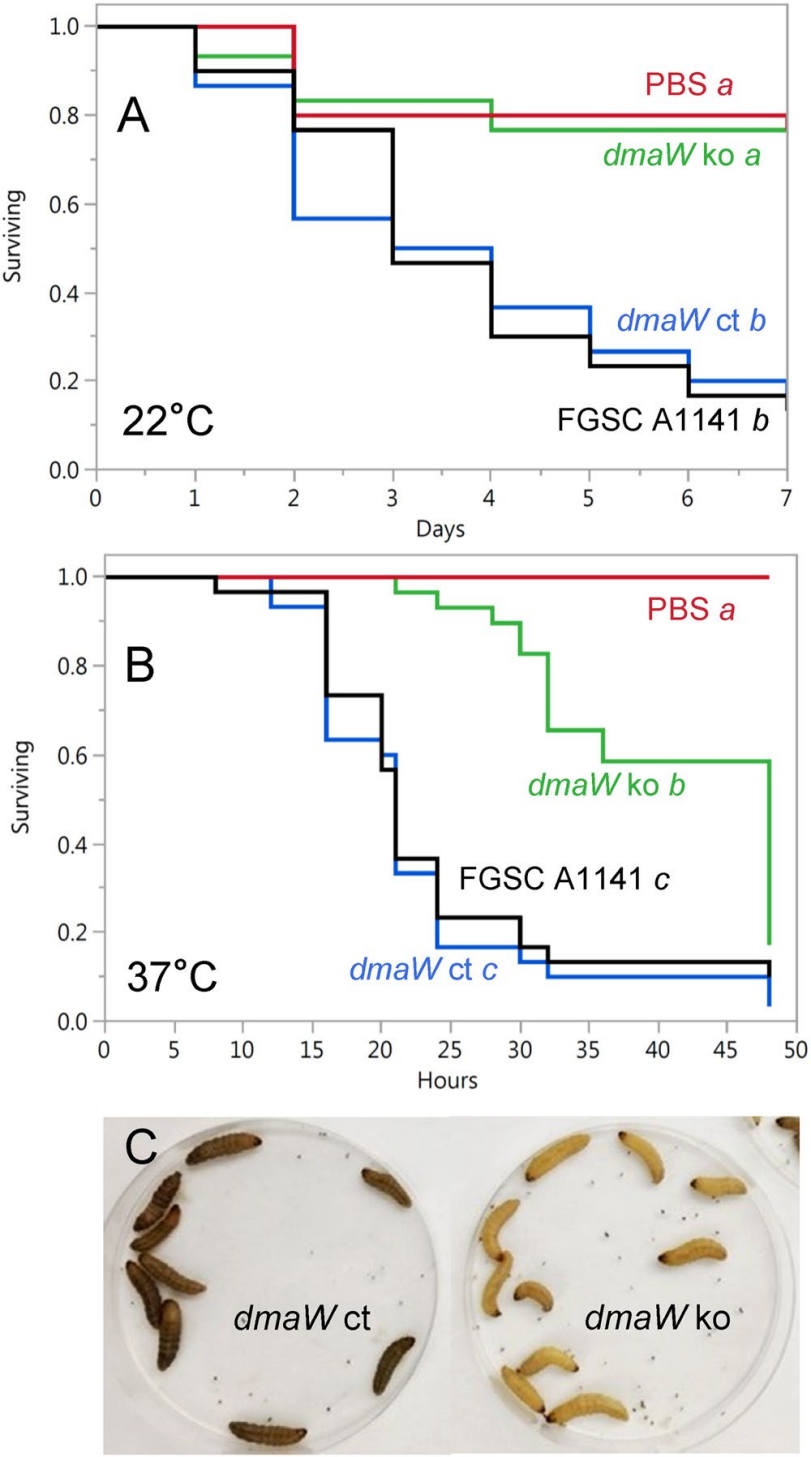
Survival curves for *G. mellonella* larvae injected with conidial suspensions of *N. fumigata* FGSC A1141, an ergot alkaloid-deficient *dmaW* knockout (ko) mutant of that strain, an ergot alkaloid restored strain complemented with a wild-type allele of *dmaW* (*dmaW* ct), or phosphate-buffered saline control (PBS). In panel A, inoculated larvae were incubated at 22 °C for seven days. In panel B, inoculated larvae were incubated at 37 °C for 48 hours. Curves were derived from data from three trials with 10 larvae per treatment per trial. Treatment labels followed by different italicised letters have survival curves that differ significantly in a log-rank test with Bonferroni-adjusted alpha value set at 0.0083 (derived from 0.05/six pairwise comparisons). Panel C shows larvae incubated at 22 °C for 5 days in one trial.

Because the ergot alkaloid pathway gene knockout strains available for study were prepared previously in two different genetic backgrounds of *N. fumigata* (Table 1), data were collected and analysed separately for the two sets of modified strains. Comparison of survival data from the *dmaW* knockout (a mutant that lacks all ergot alkaloids^38^; Table 2) and its wild-type, parental isolate *N. fumigata* FGSC A1141 indicated that ergot alkaloids were required for full virulence of *N. fumigata* in *G. mellonella*. When experiments were conducted at 22 °C, the rate of mortality caused by the *dmaW* knockout strain was significantly lower than that caused by the wild-type strain FGSC A1141 (*P* < 0.0001; Fig. 2A). Ectopic complementation with a functional allele of *dmaW* restored full virulence, demonstrating the change in virulence was due to the *dmaW* mutation (Fig. 2). The virulence of the *dmaW* knockout strain was reduced to the point where it was not statistically different from that of the PBS control (*P* = 0.94), but larvae infected with *dmaW* knockout appeared to be more strongly melanised and more lethargic than did larvae injected with the PBS control. To further investigate this issue, larvae were challenged with the same set of treatments but incubated at 37 °C (a temperature more favorable for *N. fumigata*) following inoculation. The *dmaW* knockout strain was again significantly less virulent than the parent strain FGSC A1141 (*P* < 0.0001) (Fig. 2B), but under these conditions the *dmaW* knockout strain was intermediate in virulence between FGSC A1141 and the sterile PBS control (Fig. 2B). These data demonstrate that factors other than ergot alkaloids also contribute to virulence of *N. fumigata*. As in the room-temperature experiment, the strain complemented with the functional *dmaW* allele did not differ significantly in virulence compared to its parent strain FGSC A1141 (*P* = 0.54). The times required for each strain to kill 50% of inoculated larvae (i.e., LT_50_ values) were calculated as 21 hr for both FGSC A1141 and the *dmaW* complemented strain and 48 hr for the *dmaW* knockout strain.

**Table 1:** Strains of *Neosartorya fumigata* included in this study.

Strain	Source/derivation	Most abundant ergot alkaloid(s)	Reference
FGSC A1141	Clinical	festuclavine/fumigaclavine C	45
*dmaW* ko	Gene knockout in FGSC A1141	no ergot alkaloids	38
*dmaW* ct	Complemented *dmaW* ko in FGSC A1141	festuclavine/fumigaclavine C	38
Af293	Clinical	festuclavine/fumigaclavine C	50
*easM* ko	Gene knockout in Af293	festuclavine	41
*easA*/*G* ko	Gene knockout in Af293	chanoclavine-I	current study

**Table 2:** Mean ergot alkaloid concentrations (μM; mean ± standard error) in *Neosartorya fumigata* conidial extracts injected into *Galleria mellonella* larvae.

Strain^a^	Background	Chanoclavine-I	Festuclavine	Fumigaclavine C	Summed ergot alkaloids
FGSC A1141	FGSC A1141	n.d.^b^	6.1 ± 1.2	2.0 ± 0.7	8.1 ± 1.5
*dmaW* ko	FGSC A1141	n.d.	n.d.	n.d.	n.d.
*dmaW* ct	FGSC A1141	n.d.	2.1 ± 0.4	1.1 ± 0.4	3.2 ± 0.7
filtrate	FGSC A1141	n.d.	1.0 ± 0.1	0.1 ± 0.03	1.1 ± 0.2
Af293	Af293	n.d.	1.8 ± 0.8	1.0 ± 0.5	2.9 ± 1.3
*easA*/*G* ko	Af293	0.3 ± 0.03	n.d.	n.d.	0.3 ± 0.03
*easM* ko	Af293	n.d.	9.3 ± 1.8	n.d.	9.3 ± 1.8

^a^ko, knockout; ct, complemented; filtrate, conidia removed by filtration prior to addition of methanol.

^b^n.d., not detected; limit of detection 0.07 μM.

### Larval response to conidium-free fungal extracts

**Figure 3:**
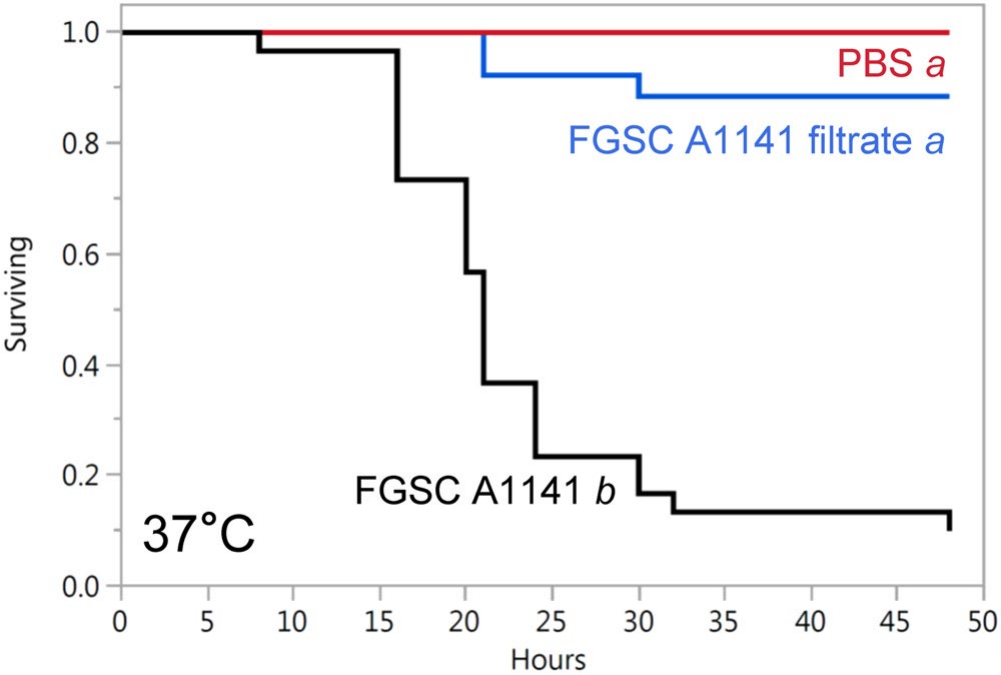
Survival curves for *G. mellonella* larvae injected with conidial suspensions of *N. fumigata* FGSC A1141, conidium-free, filtered suspensions of FGSC A1141, or phosphate-buffered saline control (PBS). Curves were derived from data from three trials with 10 larvae per treatment per trial. Treatment labels followed by different italicised letters have survival curves that differ significantly in a log-rank test with Bonferroni-adjusted alpha value set at 0.016 (derived from 0.05/three pairwise comparisons).

To test whether ergot alkaloids or other soluble factors in the *N. fumigata* FGSC A1141 conidial suspension solution were insecticidal in the absence of the fungus, 20 μL aliquots of conidial suspension solution that had been filtered through a 0.2-μm filter were assayed in *G. mellonella* larvae. The filtrate lacking conidia did not induce significantly greater mortality than did the PBS control (*P* = 0.06, with Bonferroni-adjusted alpha at 0.016). The filtered, conidium-free extract induced significantly less mortality than the unfiltered conidial suspension (*P* < 0.0001) (Fig. 3). These data indicate that mortality resulted primarily after infection with live conidia in the presence of ergot alkaloids and not as a result of acute toxicity of the conidial suspension solution. Comparison by HPLC of ergot alkaloid concentrations in *N. fumigata* FGSC A1141 conidial suspensions with filtrate prepared from those conidial suspensions indicated that only 12% of the ergot alkaloids were soluble in the filtrate, whereas the remainder must have been associated with the conidia themselves (Table 2). The observed percentage of water-soluble ergot alkaloids was consistent with previously published data^42^.

### Virulence of mutants accumulating different ergot alkaloid pathway intermediates

**Figure 4:**
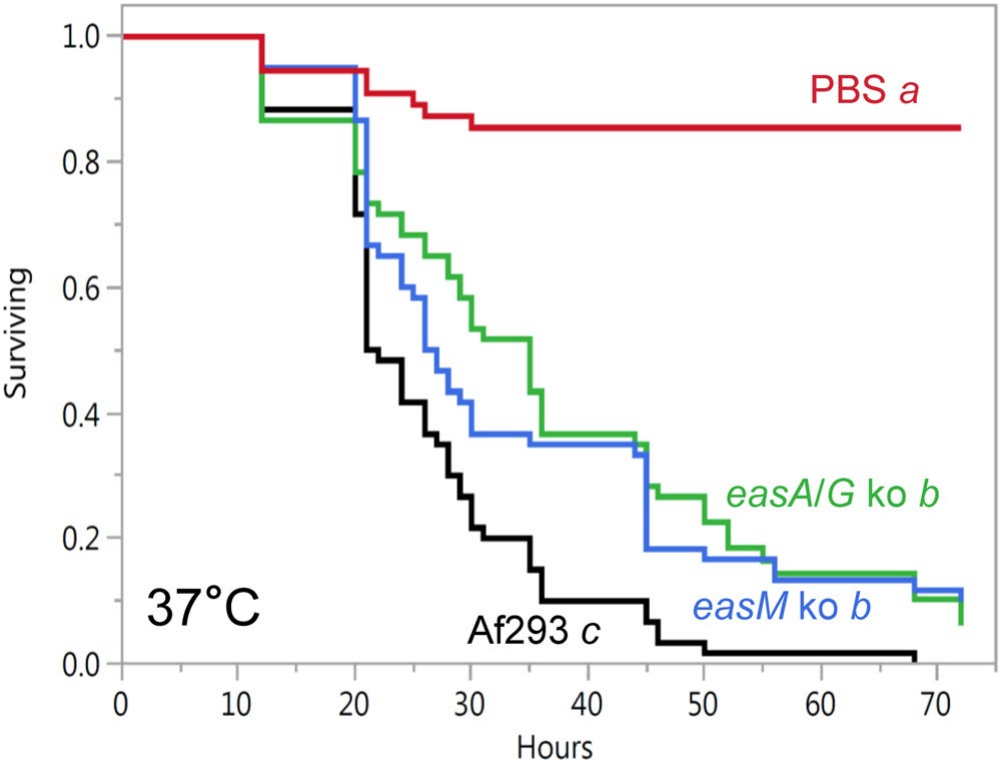
Survival curves for *G. mellonella* larvae injected with conidial suspensions of *N. fumigata* Af293, an *easM* knockout (ko) of strain Af293, an *easAG* ko of strain Af293, or phosphate-buffered saline control (PBS). Curves are derived from data from four trials with 15 larvae per treatment per trial. Treatment labels followed by different italicised letters have survival curves that differ significantly in a log-rank test with Bonferroni-adjusted alpha value set at 0.0083 (derived from 0.05/six pairwise comparisons).

Ergot alkaloid mutants in the *N. fumigata* Af293 background also demonstrated reduced virulence. An *easA*/*G* knockout strain, which is blocked after chanoclavine-I aldehyde (Fig. 1; Supplementary Information), and an *easM* knockout strain, which is blocked after festuclavine (Fig. 1), were significantly less virulent in log-rank tests than their parent strain Af293 (*P* < 0.003) (Fig. 4). These data indicate that ergot alkaloids downstream of the mutated genes were required for full virulence. LT_50_ values for the *easA*/*G* and *easM* mutant were 30 hr and 27 hr, respectively, whereas the LT_50_ value for Af293 was 21 hr. The lack of significant difference between the survival curves obtained with the *easA*/*G* knockout strain and the *easM* knockout strain (*P* = 0.32) indicates that festuclavine does not contribute significantly to mortality of larvae, or that the increase in chanoclavine-I detected in the *easA*/*G* knockout strain (Table 2) compensated for the loss of festuclavine. This result is underscored by the great abundance of festuclavine present in the *easM* knockout (Table 2).

### Correlation of mortality with concentrations of individual ergot alkaloids

**Figure 5:**
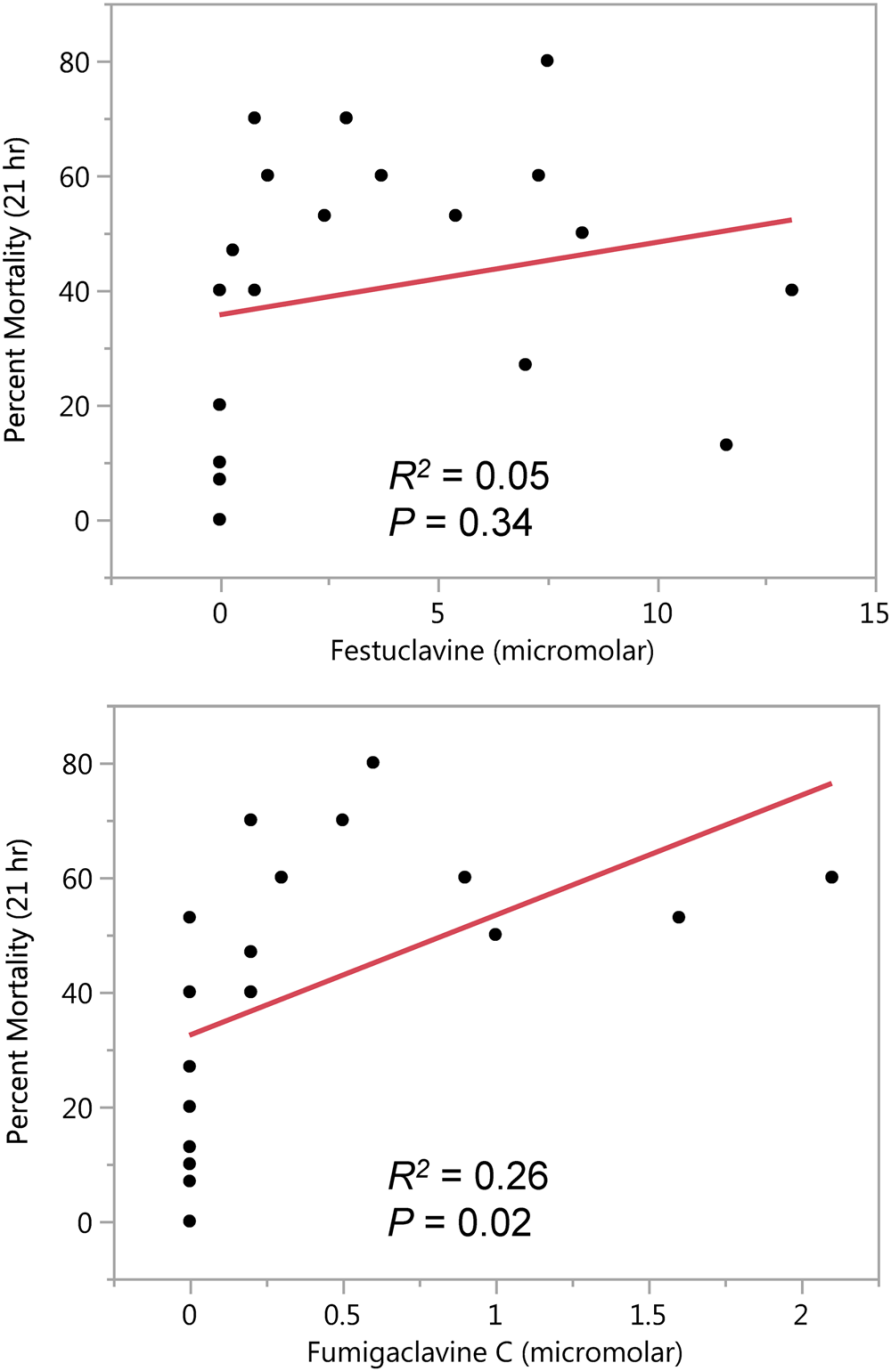
Correlation of ergot alkaloid concentrations in individual *N. fumigata* conidial extracts with larval mortality resulting from injection of that extract. Mortality was assessed at 21 hours post inoculation because both wild-type strains (Af293 and FGSC A1141) required 21 hours to kill 50% of inoculated larvae when those larvae were incubated at 37 °C. Data points represent all trials conducted at 37 °C with Af293 and FGSC A1141 and the ergot alkaloid mutants derived from those strains.

To further investigate the relative contributions of the abundantly accumulating intermediate festuclavine compared to the pathway end product fumigaclavine C, we correlated the concentrations of ergot alkaloids in each separate spore suspension sample with the percentage of larvae killed at 21 hr by injection of that same sample. This particular time point was selected because 21 hr was the LT_50_ value for both wild-type strains (Af293 and FGSC A1141) and the *dmaW*-complemented strain in experiments conducted with 37 °C incubations. The concentration of fumigaclavine C in individual samples correlated positively with larval mortality (*P* = 0.02) (though the *R* ^2^ value is low, indicating a linear response is not the best fit for the data), whereas the concentration of festuclavine did not correlate with mortality (Fig. 5). When correlation analyses were repeated with data with Af293-derived strains and FGSC A1141-derived strains tested separately, the correlation of fumigaclavine C with percent mortality was still evident (*P* = 0.04 for Af293-derived strains, and *P* = 0.06 for FGSC A1141-derived strains), whereas festuclavine did not correlate well with percent mortality (*P* = 0.78 for Af293-derived strains, and *P* = 0.10 for FGSC A1141-derived strains).

## Discussion

Disruption of the ergot alkaloid pathway in two different genetic backgrounds reduced the virulence of the opportunistic human pathogen *N. fumigata*, when tested in the invertebrate model *G. mellonella*. Since we made use of existing mutants, one of the mutants tested (*dmaW* knockout) was in a *N. fumigata* FGSC A1141 background, whereas two other mutants (*easA*/*G* knockout and *easM* knockout) were in a *N. fumigata* Af293 background. Both parent strains produce fumigaclavine C as their pathway end point and have similar ergot alkaloid profiles^43^ but presumably contain other genetic differences. Because of this difference in backgrounds, we did not compare the survival curves of the *dmaW* knockout with the *easM* and *easA*/*G* knockouts by a log-rank test. Previous studies have shown that different environmental isolates vary in their virulence^30, 31, 44^. Adhering to within-isolate limits, we can conclude that elimination of all ergot alkaloids from *N. fumigata* isolate FGSSC A1141 significantly reduced virulence and that elimination of alkaloids downstream from chanoclavine-I aldehyde and festuclavine in strains derived from *N. fumigata* Af293 also reduced virulence. The abundantly accumulating intermediate festuclavine (which was the end product of the pathway in the *easM* knockout) did not appear to be a significant virulence factor because the *easM* knockout (which accumulated high concentrations of festuclavine) did not differ in virulence from the *easA*/*G* knockout whose pathway was blocked prior to festuclavine. Moreover, larval mortality in individual trials did not correlate with festuclavine concentration but did correlate significantly with concentration of fumigaclavine C, which appears to be a significant virulence factor.

The effect of temperature on mortality rate among strains is probably multifaceted. The observation that the *dmaW* knockout strain appeared more debilitated at ambient temperature as opposed to 37 °C may have resulted from the effects of temperature on fungal growth rate, production of undetermined fungal metabolites or enzymes, the insect’s immune system, or a combination of these factors. Certainly, the increase in temperature to 37 °C favored the fungus from a growth-rate perspective. Previous studies with *C. neoformans* also documented an increased mortality rate at 37 °C as opposed to 30 °C^33^. Irrespective of the cause, at the higher temperature the residual virulence of the ergot alkaloid-deficient, *dmaW* knockout strain was distinguishable from the background mortality associated with injection of the PBS control.

The approach to ergot alkaloid analysis that we took in this current study enabled us to document the concentrations of ergot alkaloids in each individual inoculum sample; however, this approach limited us to detecting only the most abundant ergot alkaloid in each sample. Ergot alkaloids are closely associated with conidia of *N. fumigata* ^42, 45, 46^ and typically are extracted with methanol to acquire sufficient quantities for accurate quantification^47^. In this present study, ergot alkaloids were extracted with 50% methanol from aqueous conidial suspensions. Use of an initially aqueous solution was necessary to maintain viability of conidia that were to be used as inoculum and to avoid toxicity of injecting an organic solvent into larvae. A second major limitation on our ability to detect and quantify ergot alkaloid intermediates in this present study was that we extracted only 50,000 conidia per µL (derived from the concentration injected into larvae), whereas extracts in our previously published analyses typically contained ten to 100 times more conidia per µL. As a result of these constraints on alkaloid analysis, roles for other intermediates and alternate products of the ergot alkaloid pathway are more difficult to assess. Other ergot alkaloids were probably present in the injected conidial suspension but were below the limit of detection. Ergot alkaloid pathways in general are inefficient in that several intermediates typically accumulate to measurable levels^48^. Accumulation of several ergot alkaloid pathway intermediates including chanoclavine-I, fumigaclavine A, and fumigaclavine B have been measured in *N. fumigata* strains Af293 and FGSC A1141^38, 43^.

Previous studies with *N. fumigata* and *G. mellonella* provided indirect evidence of the importance of fumigaclavine C in virulence^30, 31^. In these studies, a gene knockout strain deficient in peptide synthetase PesL had reduced virulence to *G. mellonella* ^30^, whereas the contribution of peptide synthetase Pes1 to virulence varied by genetic background of the knockout strain^30, 31^. The exact functions of these peptide synthetases are unknown but, unexpectedly, both appear to be required for the final step in the biosynthesis of fumigaclavine C in *N. fumigata* ^30^. The data of O’Hanlon *et al*.^30^ suggest that the lack of virulence in the PesL mutant resulted at least in part from the lack of fumigaclavine C. The role of Pes1 in virulence was less clear, since knockout of *pes1* in an Af293.1 background reduced virulence in the *G. mellonella* model, but knocking out this same gene in an ATCC 46645 background did not significantly reduce virulence. The effects of Pes1 and PesL on fumigaclavine C biosynthesis could not be separated from the effects of these peptide synthetases on other metabolites and processes (such as increased sensitivities to oxidative stress and antifungal compounds^30, 31^). Interestingly, earlier steps in the ergot alkaloid pathway appeared to be unaffected by the *pesL* mutation^30^, indicating that alkaloids upstream of fumigaclavine C were less important for virulence.

The data presented herein provide direct evidence of a role for ergot alkaloids in virulence of *N. fumigata* to animals. Ergot alkaloids are well known for their bioactivity in mammals and have been exploited as pharmaceuticals and illicit drugs. They are best known from plant-associated fungi in the Clavicipitaceae (*Claviceps* species and *Epichloë* species) where they affect herbivores, but the alkaloids have no known effects on the plants themselves^5, 6^. Ergot alkaloids such as lysergic acid derivatives produced by *Epichloë* species do have anti-insect activities upon ingestion^14, 49^, but the ergot alkaloids of *N. fumigata* did not induce significant mortality upon injection at the concentrations used in this present study. Since *N. fumigata* has no mechanism for making ingress into an insect, it is unlikely that its ergot alkaloids affect insects in nature. The means by which ergot alkaloids contribute to animal pathogenesis is not known, but based on their close association with conidia one potential mechanism would be protection of inhaled conidia from cells of the host’s innate immune system.

## Methods

### Experimental organisms

Two wild-type isolates of *N. fumigata* (FGSC A1141 and Af293) that contained a complete functional pathway to fumigaclavine C^43^ and ergot alkaloid mutants derived from these wild-type isolates were studied. All fungi were cultivated on malt extract agar (per L: 6 g malt extract, 1.8 g maltose, 6 g dextrose, 1.2 g yeast extract, 15 g agar). The *dmaW* knockout (strain FGSC A1141 background), which lacks the capacity to produce any ergot alkaloids, and its ectopically complemented derivative were characterised previously^38^. Two ergot alkaloid mutants in the *N. fumigata* Af293 background also were studied. Strain *easM* knockout contains a replacement in the gene *easM* which encodes a P450 monooxygenase required to oxidise festuclavine to fumigaclavine B^41^; as a result, the *easM* knockout accumulates large quantities of festuclavine and lesser quantities of chanoclavine-I. Strain *easA*/*G* knockout contained a replacement of a fragment that spanned adjacent genes *easA* and *eas*G (Supplementary Information). Since *easA* immediately precedes *easG* in the ergot alkaloid pathway, the *easA*/*G* knockout mutant has the phenotype of the previously characterised *easA* knockout^39^, meaning that it accumulates smaller quantities of chanoclavine-I aldehyde and larger quantities of the immediate precursor chanoclavine-I^39, 40^.

*Galleria mellonella* larvae were purchased from a local franchise of Petco and were inoculated the same day they were purchased. Larvae that were active, light colored (without any dark markings), and weighed at least 200 mg were chosen for inoculation.

### Pathogenicity assays

Pathogenicity assays were derived from methods described previously^28, 32–36^. Conidia from cultures of the indicated strains grown on malt extract agar for two to four weeks were harvested by tapping an inverted culture such that large numbers of conidia collected on the inside of the Petri dish lid. Conidia were suspended in a modified PBS solution (pH 7.4) containing 9 mM Na_2_PO_4_, 1.6 mM KH_2_PO_4_, 123 mM NaCl, 0.01% wt/vol tween 20, and 10 μg/mL rifampin. Spore concentration was determined with the aid of a hemocytometer by counting dilutions made in 0.1% aqueous tween 20. Concentrations were adjusted with the modified PBS solution to obtain final concentrations of 2.5 × 10^5^ conidia/μL for experiments conducted at 22 °C and 1 × 10^5^ conidia/μL for experiments conducted at 37 °C. Once the desired conidial concentration was obtained, 20 μL of conidial suspension (containing 5 million conidia for experiments at 22 °C or 2 million conidia for experiments at 37 °C) was injected with a disposable 29.5-gauge hypodermic needle through the hindmost left proleg of a *G. mellonella* larva. A fresh hypodermic needle and syringe was used for each treatment. To test for acute toxicity of ergot alkaloid-containing conidial suspensions, aliquots of conidial suspensions of FGSC A1141 were spin-filtered through a 0.2-μm nylon filter to remove conidia prior to injection. Ten larvae per treatment were injected with each treatment or control for experiments involving strains derived from FGSC A1141, and 15 larvae per treatment were injected for experiments involving strains derived from *N. fumigata* Af293. Experiments involving strains derived from FGSC A1141 were repeated three times at 22 °C and three times at 37 °C, whereas those involving Af293-derived strains were repeated four times at 37 °C. Potential sources of variation included batch-to-batch differences in quality of *G. mellonella* larvae, quantification of hydrophobic spores in an aqueous solution, and quality of conidia and quantities of alkaloids present when harvesting conidia from cultures of slightly different ages. For these reasons, each experiment was conducted three times (for FGSC A1141 derivatives) or four times (for Af293 derivatives) with fresh batches of larvae and freshly-prepared conidial suspensions in each trial, and all results were included. Mortality was assessed by failure of larvae to move in response to being rolled over their back and typically was accompanied by melanisation. Observations were made twice daily for trials run at 22 °C and every two to eight hours for trials conducted at 37 °C. Slight differences in the timing of observations among trials resulted in stair-steps at unequal intervals along the x axis of Kaplan-Meier plots.

### Statistical analyses

Results were analysed by Kaplan-Meier survival plots, and differences among survival curves were tested by log-rank tests. To compensate for multiple pairwise comparisons in a given experiment, Bonferroni corrections were applied such that the critical alpha value (0.05) was divided by the number of comparisons made in each experiment. The Bonferroni-adjusted alpha value is indicated in the legends of relevant figures. To correlate larval mortality with concentrations of certain ergot alkaloids in injected conidial suspensions, percent mortality values for all fungal treatments incubated at 37 °C were correlated with molar concentrations of individual ergot alkaloids in each injected conidial suspension. Mortality was assessed at 21 hr in these correlation analyses, because that was the time required for both wild-type isolates to kill 50% of inoculated larvae. When fungal isolate genetic background (*i.e*., Af293 vs. FGSC A1141 as origin of strains) was considered as a factor in ANOVA, background did not have a significant effect (*P* = 0.48) on percent mortality at 21 hr; thus, data from both backgrounds were included in the correlation analyses. All statistical analyses were performed with JMP (SAS, Cary, NC, USA). Data are available upon request to the corresponding author.

### Analysis of ergot alkaloids

Ergot alkaloids in each extract were assayed by diluting the extract with an equal volume of HPLC-grade methanol, incubating at room temperature for 30 min, and centrifuging for 5 min to remove conidia and any precipitate prior to analysis by high performance liquid chromatography (HPLC). Ergot alkaloids were analysed by HPLC with fluorescence detection as described previously^47^. Briefly, 20 μL of alkaloids were separated on a reverse-phase C18 column (Phenomenex Prodigy, Torrance, CA, USA; 5-μm particle size ODS3, 150 mm × 4.6 mm) with a multi-linear, binary gradient of from 5% (vol/vol) to 75% acetonitrile in aqueous 50 mM ammonium acetate. Analytes were monitored by fluorescence with excitation wavelength set at 272 nm and emission wavelength set at 372 nm.

## Electronic supplementary material

Supplementary Information
