# Supplementary material for: Ergot alkaloids contribute to virulence in an insect model of invasive aspergillosis
Source: Sci Rep. 2017 Aug 21;7:8930. doi: 10.1038/s41598-017-09107-2 (PMC5567044; doi:10.1038/s41598-017-09107-2)
Supplement: Supplementary file 1 — Supplementary Information [file 41598_2017_9107_MOESM1_ESM.pdf]

Supplementary Information

Title: Ergot alkaloids contribute to virulence in an insect model of invasive aspergillosis

Author list: Daniel G. Panaccione and Stephanie L. Arnold

Table S1. PCR primers and products

Primer combination	Primer sequences 5′→3′	Product description	Amplicon length (bp)
gAf +<br>pAr	GAGAACCTTGGTGAGTTGCCC +<br>GTTAGCTCACTCATTAGGCACC	Bridges integration border on *easA* side	2396
pGf +<br>gGr	GATGGCCCACTACGTGAACC +<br>CTGTGGATGCGACTATTCTCC	Bridges integration border on *easG* side	1892
*alb*F +<br>*alb*R	GGTCGGCGCTCCTCAAATGG +<br>GGATATCCTTCACGCTCGACC	Part of *N. fumigata alb1* gene as template positive control	1595

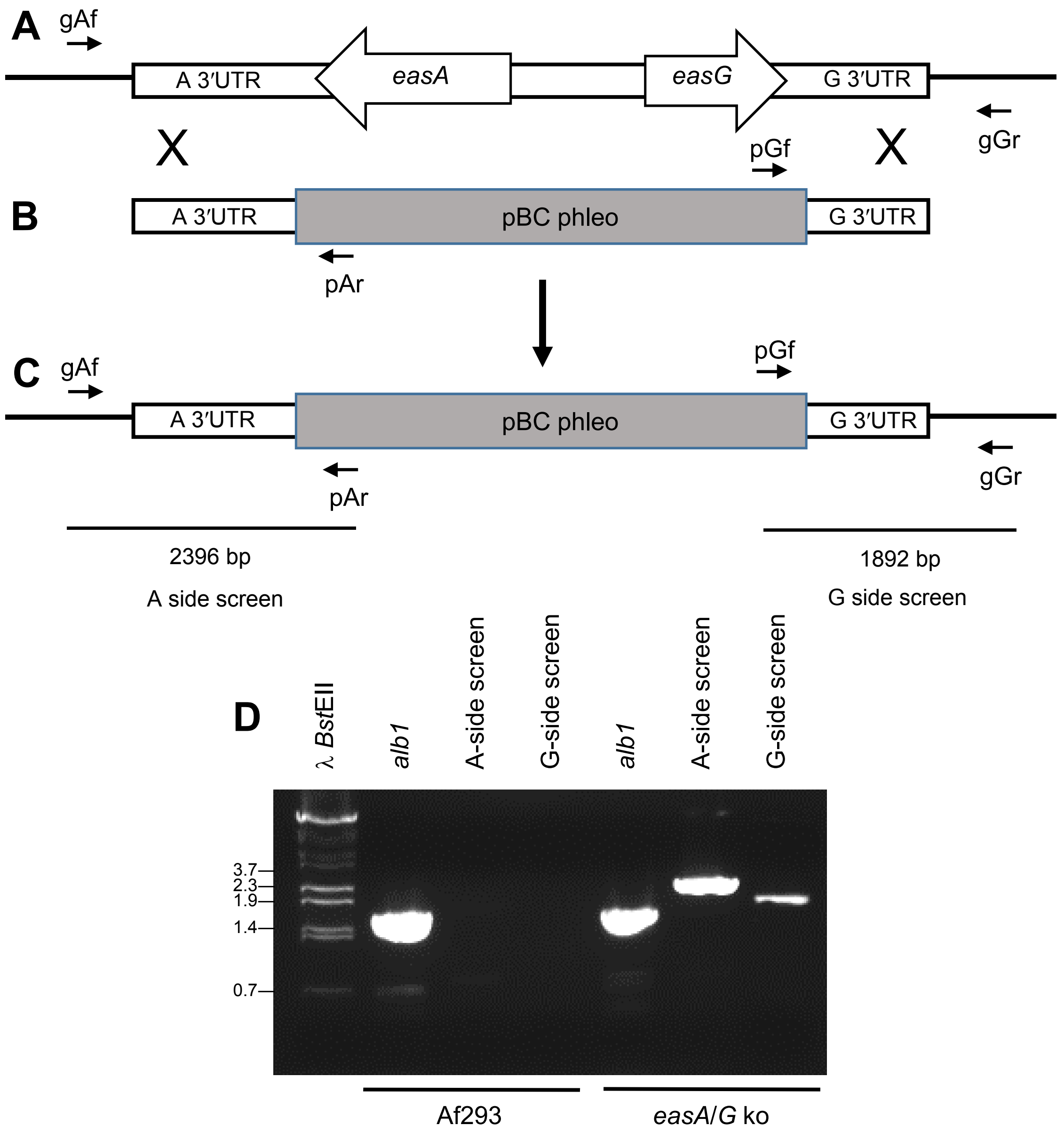

Fig. S1. Construction and verification of *easA*/*G* knockout in *N. fumigata* Af293. (A) Region of the *N. fumigata eas* cluster targeted for recombination is shown as a white rectangle. Position and orientation of coding sequences of *easA* and *easG* are indicated with enlarged arrows. The 3′ untranslated regions of *easA* (A 3′UTR) and *easG* (G 3′UTR) targeted for recombination are labelled. (B) Gene knockout construct containing 1997 bp of *easA* 3′UTR (coordinates 4774858-4776855 on Af293 chromosome 2; accession NC_007195) and 1299 bp of *easG* 3′UTR (coordinates 4780007-4781306 on Af293 chromosome 2; accession NC_007195) attached at *Cla*I and *Spe*I sites, respectively, of pBC-phleo (Silar[1]; Fungal Genetics Stock Center, Manhattan, KS, USA). (C) Homologous recombination of the construct replaces *easA* and *easG* with pBC-phleo and brings primer annealing sites for indicated primers (Table S1) within specified distances of one another. (D) PCR products derived from template DNA from Af293 or an *easA*/*G* knockout strain with indicated primer pairs (Table S1). The *alb1* gene served as a positive control for amplifiable template DNA. Primer annealing sites for A side screen and G side screen are only near each other in the knockout strain. Lengths of relevant fragments of *Bst*EII-digested bacteriophage lambda DNA are indicate at left. Illustrated features in panels A, B, and C are not drawn to scale.

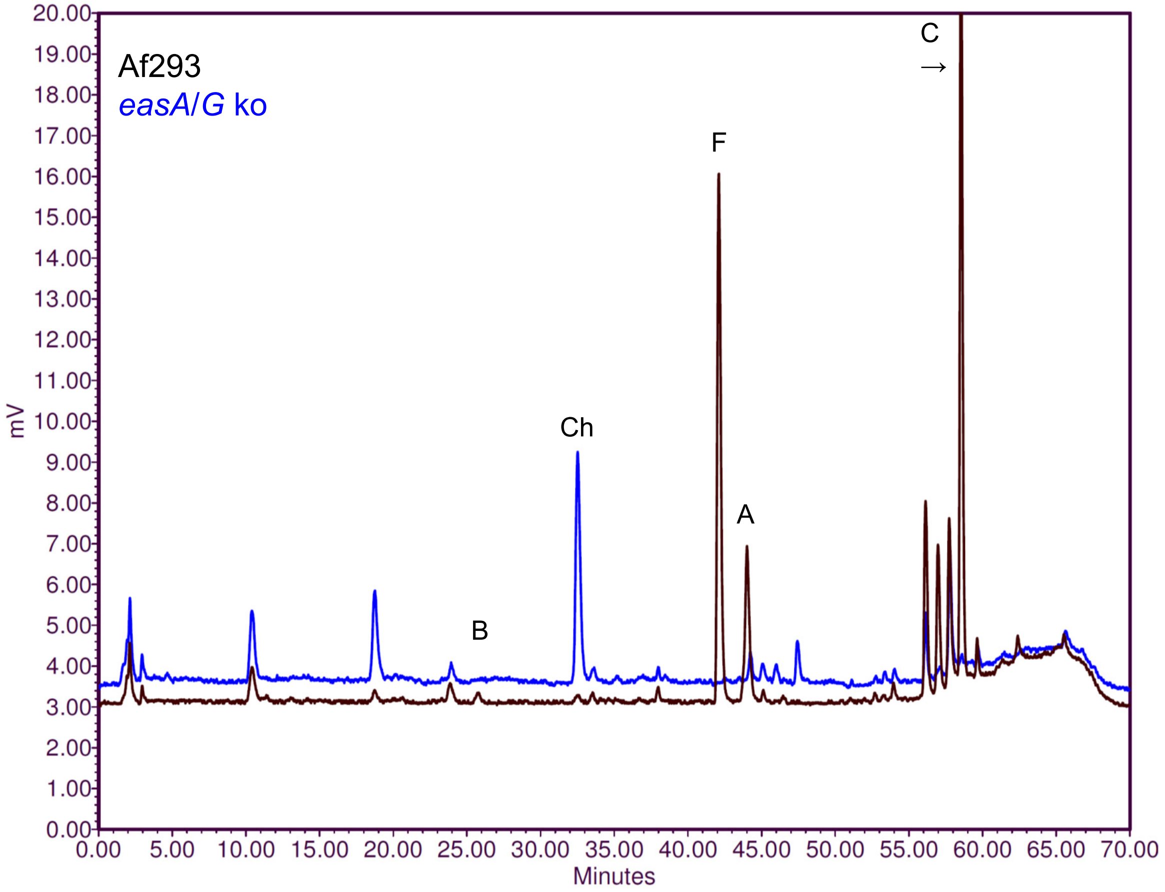

Fig. S2. Analysis of *easA/G* knockout of *N. fumigata* Af293 by high performance liquid chromatography (HPLC) with fluorescence detection. Alkaloids were extracted with HPLC-grade methanol from a plug of a malt extract agar culture containing 50-mm^2 of surface area. Fluorescence was monitored at 372 nm after exciting at 272 nm according to described methods[2]. The chromatogram from the *easA/G* knockout strain is coloured blue, whereas that from the parent strain Af293 is black. Peaks corresponding to characterized ergot alkaloids are indicated with the following abbreviations: B, fumigaclavine B; Ch, chanoclavine-I; F, festuclavine; A, fumigaclavine A; and, C, fumigaclavine C. The data are consistent with the phenotype of the *easA* knockout of *N. fumigata* described previously[3, 4].

References for Supplementary Information

1. Silar, P. Two new easy to use vectors for transformations. *Fungal Genet. Rep.* **42,** 73 (1995).

2. Panaccione, D. G., Ryan, K. L., Schardl, C. L. & Florea, S. Analysis and modification of ergot alkaloid profiles in fungi. *Methods Enzymol*. **515,** 267-290 (2012).

3. Coyle, C. M., Cheng, J. Z., O'Connor, S. E. & Panaccione, D. G. An old yellow enzyme gene controls the branch point between *Aspergillus fumigatus* and *Claviceps purpurea* ergot alkaloid pathways. *Appl*. *Environ*. *Microbiol*. **76**, 3898-3903 (2010).

4. Cheng, J. Z., Coyle, C. M., Panaccione, D. G. & O'Connor, S. E. A role for old yellow enzyme in ergot alkaloid biosynthesis. *J. Amer. Chem. Soc.* **132**, 1776-1777 (2010).
